# Cognitive Rehabilitation in Multiple Sclerosis: The Role of Plasticity

**DOI:** 10.3389/fneur.2015.00067

**Published:** 2015-04-02

**Authors:** Nancy D. Chiaravalloti, Helen M. Genova, John DeLuca

**Affiliations:** ^1^Neuropsychology and Neuroscience Laboratory, Kessler Foundation, West Orange, NJ, USA; ^2^Department of Physical Medicine and Rehabilitation, Rutgers New Jersey Medical School, Newark, NJ, USA; ^3^Department of Neurology and Neurosciences, Rutgers New Jersey Medical School, Newark, NJ, USA

**Keywords:** multiple sclerosis, cognitive rehabilitation, neuroimaging, fMRI, cognitive remediation, cognition

## Abstract

Cognitive deficits are common in multiple sclerosis (MS), documented at many stages of the disease. Both structural and functional neuroimaging have demonstrated a relationship with cognitive abilities in MS. Significant neuroplasticity of cognitive functions in individuals with MS is evident. Homologous region adaptation, local activation expansion, and extra-region recruitment all occur in an effort to maintain cognitive functioning. While much of this neuroplasticity is adaptive, it may also be maladaptive, particularly in individuals that are demonstrating significant cognitive impairment and/or with disease progression. This maladaptive neuroplasticity may come at the cost of other cognitive functions. Studies of cognitive rehabilitation efficacy have also recently applied neuroimaging techniques to establish outcome. Researchers have successfully applied various neuroimaging techniques to study the effects of cognitive rehabilitation in MS including task-based fMRI and resting state functional connectivity across multiple realms of cognition including episodic memory, executive functioning, attention, and processing speed. These studies have demonstrated neuroplasticity in the brains of persons with MS through the documentation of changes at the level of the cerebral substrate from before to after non-invasive, non-pharmacological, behavioral treatment for deficits in cognition. Future research should seek to identify adaptive versus maladaptive neuroplasticity associated with specific cognitive rehabilitation programs within all MS phenotypes to foster the validation of the most effective cognitive rehabilitation interventions for persons with MS.

## Cognitive Rehabilitation in Multiple Sclerosis: The Role of Plasticity

Multiple sclerosis (MS) is a progressive neurological disease marked by the development of lesions, or plaques, throughout the brain and spinal cord. The disease has been shown to impact both the white and gray matter of the brain often resulting in permanent disability (1–3). A broad array of symptoms is common in persons with MS, including motor, psychiatric, and cognitive symptomatology (4).

Cognitive deficits are common in MS, with prevalence rates ranging from 43 to 70% (5–7). MS impacts multiple aspects of cognition and may appear either early or late in the disease process. Deficits in information processing speed represent the most common cognitive deficit in MS (8–13). Other prevalent areas of deficit include attention (13, 14), executive functioning (15–17), working memory (18, 19), and long-term memory (4, 20–23). Overall intellectual functioning generally remains intact (24), as do “simple” attention (i.e., repeating numbers) and basic verbal skills (i.e., word naming, comprehension)(25). The clinical presentation is thus typically one of cognitive deficits, sometimes mild to moderate in nature, impacting specific cognitive domains. Due to the fact that the cognitive profile in MS is generally not one of a generalized dementia, cognitive rehabilitation is particularly appropriate for persons with MS. Cognitive deficits can often be specifically identified through a comprehensive neuropsychological assessment and subsequent cognitive rehabilitation can target discrete areas of dysfunction in an effort to improve overall cognitive abilities and quality of life (QoL).

Cognitive dysfunction has been shown to exert a significant negative impact on the every day lives of persons with MS. Persons with MS with cognitive impairment participate in fewer social and vocational activities (25), have higher rates of unemployment or under employment (5, 25–28) and show greater difficulties in doing routine household tasks (25, 29). Deficits in new learning and memory in particular have been shown to result in a reduced ability to make decisions that could affect functioning in everyday life (30) and negatively impact daily living (31–33). Common resultant functional impairments include difficulty with household chores, shopping, completing home repairs, driving, and using public transportation (34, 35). Reduced QoL is often reported (36).

Given the significant impact of cognitive deficits on the everyday lives and overall QoL of persons with MS, it is imperative that we develop and validate mechanisms for effectively treating cognitive dysfunction in this population. Numerous studies have demonstrated cognitive rehabilitation to be effective across many domains of functioning in other neurological populations. For instance, recent systematic reviews have shown that cognitive interventions can significantly improve functioning in persons with TBI and stroke (37–39). Cognitive rehabilitation has also led to significant gains in the aging population across both objective neuropsychological performance (40) and the performance of daily life activities (41–43), with effects maintained up to 10 years post-treatment (44). There have, however, been considerably fewer studies on cognitive rehabilitation in MS, with many of these studies suffering from significant methodological difficulties (45–48). More recent, well-designed studies have been more promising and have provided evidence of improved objective cognitive performance as well as improvements in everyday life activities following cognitive rehabilitation [e.g., (45, 49–51)].

### Cognitive rehabilitation in MS: The role of neuroimaging

The term *neuroplasticity* refers to “the ability of the nervous system to respond to intrinsic and extrinsic stimuli by reorganizing its structure, function, and connections” [(52); p. 1591]. That is, the brain is able to reorganize its structural and functional connections in an effort to maximize functional capacity and “adjust” its resources to cope with cognitive impairments. Changes in functional activation in persons with MS have often been correlated with improved cognitive performance, such as following cognitive rehabilitation; authors have thus interpreted such neuroplasticity as having a positive or “adaptive” outcome (50, 53). However, it is important to recognize that such plasticity may also be “maladaptive.” The term “maladaptive plasticity” may be used to refer to cerebral inefficiency in situations in which such neuroplasticity is correlated with cognitive impairment or decline (54, 55).

Neuroplasticity has recently been observed in numerous studies to explain treatment efficacy of cognitive rehabilitation. That is, both structural and functional neuroimaging have been shown to be related to improvements in cognitive abilities in MS following treatment. In studies of cognition in MS utilizing neuroimaging, cognitive impairments in MS have been related to various measures of cerebral integrity including, T2 lesion load (56), cerebral atrophy (57), third ventricular width (58), corpus callosum size (59), and cortical lesions (60). In addition, the wide application of functional neuroimaging techniques to the MS population has demonstrated alterations in patterns of cerebral activation and functional connectivity. Task-based fMRI is a widely used approach to understanding the cerebral resources involved in completing a specific cognitive task. This approach affords researchers the opportunity to examine levels of activation during task performance in specific brain regions. These altered patterns of cerebral activation have been documented during tasks involving attention (61–63), working memory (54, 63–66), episodic memory (64, 65, 67), and processing speed (68). That is, fMRI studies have noted changes in the functional organization of the brain in MS patients compared with healthy individuals. In addition, studies have even noted that patients in early stages of MS activate additional regions during task performance, prior to cognitive deficits being detectable on neuropsychological assessment [e.g., Ref. (69, 70)]. It has been proposed that this additional activation serves as a compensatory mechanism allowing the individual to maintain intact cognitive functioning for a period of time (69, 71, 72). In more severely impaired patients, however, the data are less consistent. Some groups have noted activation patterns to be comparable with controls (62), despite impairments in cognitive performance, with fewer areas of increased activation than is evident in patients in the earlier stages of the disease. This pattern of findings has been interpreted as an inability to access the additional cognitive resources needed to effectively perform the task (73). Others studies have noted increased activation on task-based fMRI in cognitively impaired patients with MS (54, 55), with this increase in activation correlated with worse performance on cognitive tasks. Due to its correlation with *greater* cognitive impairment, this increased activation is deemed *maladaptive* in nature and has been interpreted as neural inefficiency.

Resting state functional connectivity (rs-FC) studies have similarly noted increased activation to be interpreted as either adaptive or maladaptive in nature, depending on the progression of the disease. In contrast to task-based fMRI, rs-FC allows the examination of the communication between different brain regions within neural networks, while at “rest.” Increased connectivity during rs-FC is thought to serve as a compensatory mechanism for cognitive deficits early in the MS disease process (71, 74–76). For example, early alterations in neuronal synchronization in rs-FC networks in clinically isolated syndrome (CIS) have been interpreted to be compensatory, indicating cortical reorganization. Such alterations may not be observed with increased brain damage, thought to indicate that such reorganization is finite and only evident early in the disease process (73). Interestingly, other work has noted that when increased rs-FC is found later in the disease process, this increase rs-FC appears to be maladaptive, similar to that which was found in some task-based fMRI studies [e.g., (54, 55)]. That is, increased rs-FC has been shown to be related to increased cognitive dysfunction in MS samples (77). Thus, early in the disease increased rs-FC appears to be adaptive, but later in the disease, these extra connections are associated with worse performance.

Functional neuroimaging techniques thus provide a means of understanding functional reorganization and neural plasticity in response to the disease process. Functional neuroimaging could similarly be used to observe neural plasticity following effective cognitive rehabilitation. The advantage of using functional neuroimaging in conjunction with traditional neuropsychological outcomes is that researchers can observe, not only the traditional behavioral improvements on cognitive tasks but also changes in the functional cerebral architecture underlying such cognitive improvements. Thus, several recent studies have utilized neuroimaging techniques to evaluate the neurofunctional and neuroanatomical changes associated with cognitive rehabilitation in MS samples. These studies have demonstrated neuroplasticity of the brain of the person with MS through the documentation of changes at the level of the cerebral substrate from before to after *non-invasive*, *non-pharmacological*, *behavioral* treatment for deficits in cognition.

Researchers have successfully applied various neuroimaging techniques to study the effects of cognitive rehabilitation in MS including task-based fMRI [e.g., (78)] and rs-FC [e.g., (79)] across multiple realms of cognition including episodic memory (78), executive functioning, attention, and processing speed (45, 50, 51). Studies have even begun to examine longer-term maintenance of such functional changes, documenting sustained plasticity over time (80).

Research conducted utilizing these neuroimaging techniques have consistently demonstrated significantly increased cerebral activity following cognitive rehabilitation [e.g., (50, 53, 78, 79, 81–83)], with numerous researchers noting induced neural plasticity in response to cognitive rehabilitation. As expected, the specific brain regions in which changes in activation patterns are documented post-treatment varies with the treatment protocol investigated, the specific cognitive function targeted for treatment, as well as the imaging protocol applied. Studies also show differences in the documentation of a relationship between these changes in patterns of cerebral activation and changes in behavior documented via neuropsychological assessment. Specifically, some studies have found that the changes on fMRI to correlate with improvement on neuropsychological assessment in the targeted domain [e.g., (50, 53)], while others have failed to document such a relationship [e.g., (84)].

**Figure 1:**
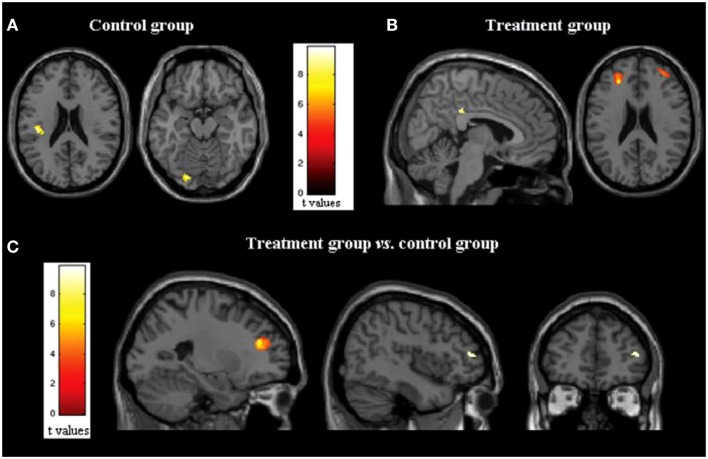
**(A,B)** Statistical parametric mapping results (color-coded for *t* values) overlaid on high-spatial resolution T1-weighted MR images show changes in functional MR imaging activations during the Stroop interference condition in **(A)** control group (axial images) and **(B)** treatment group (*p* = 0.05, paired *t* test, family-wise corrected for multiple comparisons) (sagittal and axial images). **(C)** Statistical parametric mapping results (color-coded for *t* values) overlaid on high-spatial-resolution T1-weighted MR images show between group comparisons of functional MR imaging activations during the Stroop interference condition (analysis of variance, two-by-two factorial design; *p* = 0.05, family wise corrected for multiple comparisons) in treated group versus control group (sagittal and coronal images). Here and throughout, images are in neurologic convention (i.e., left side of the image shows left side of the brain, right side of the image shows right side of the brain). *Reprinted with permission from the Journal of Radiology.

The majority of studies applying fMRI and rs-FC to the investigation of the efficacy of cognitive rehabilitation in MS have focused on the amelioration of attentional deficits. The attention/information processing modules of the cognitive therapy program, the RehaCom (85), have been far received the most attention. Filippi et al. (50) utilized both fMRI and rs-fMRI to examine the cerebral impact of cognitive retraining in 10 persons with MS that completed treatment and 10 that did not complete treatment. The treatment protocol examined consisted of a portion of the RehaCom addressing attention, information processing, and executive functioning. An improvement in cognitive functioning was noted on the Wisconsin card sorting test (WCST; a test of executive functioning), the paced auditory serial addition test (PASAT; a processing speed and working memory test), and controlled oral word association from pre to post-treatment. While no differences were noted on structural measures, the group who received the treatment showed significantly increased activation on fMRI in the posterior cingulate cortex (PCC)/precuneus and dorsolateral prefrontal cortex (DLPFC) bilaterally compared to the placebo group (Figure 1). An increase in rs-FRI was also noted after the treatment period in the treatment group only in the right PCC and the IPL of the default mode network (DMN). The DMN is a cortical network that has been shown to be active when the individual is at rest and deactivated when the individual is actively engaged in a cognitive task (86). Finally, increased rs-FC was noted in the treatment group only in the executive functioning network (left DLPFC) as well, which is implicated in active cognitive control during task performance. The increased activity in these networks was interpreted to be indicative of compensatory activation due to treatment effects. These authors also noted positive correlations between changes in rs-FC and cognitive performance, as well as changes on fMRI and cognitive performance, such that increased activation and increased rs-FC were each associated with improved task performance. This was observed across all subjects and when examining the treatment group only. Importantly, regions showing post-treatment changes in activity are areas known to be active in cognitively demanding tasks (87). Further analyses of the same data revealed increased rs-FC of the anterior cingulate cortex (ACC) as well as within the right middle frontal gyrus and the right IPL in the treatment group but not the control group (82). The control group showed decreased activation at follow-up in the ACC as well as the right cerebellum and the right inferior temporal lobule. In a follow-up investigation by the same group (88), rs-FC changes in the DMN following treatment predicted cognitive performance 6 months later. This indicates that the changes in patterns of cerebral activation and connectivity following cognitive rehabilitation can be maintained over time.

Also examining the RehaCom, Bonavita et al. (84) investigated changes in functional connectivity from before to after 8 weeks of cognitive rehabilitation with specific sections of the RehaCom program, namely, *Attention and Concentration*, *Plan a Day*, *Divided Attention*, *Reaction Behavior*, and *Logical Thinking*. They contrasted this treatment with a control group that received a placebo intervention. Post-treatment cognitive gains were noted in only the treatment group in processing speed abilities [symbol digit modalities test (SDMT) and PASAT] and verbal and visual learning and memory [(selective reminding test (SRT) and the spatial recall test (SPART-10/36)]. Changes were also noted in the DMN post-treatment, specifically increased FC in the PCC and IPC. In contrast to Filippi et al. (50), these authors failed to find a correlation between changes in FC and improvement in neuropsychological functioning.

Specifically focused on the neuroplasticity of the cerebellum, Cerasa et al. (53) demonstrated that specific, computer-based training for attention deficits results in adaptive neural plasticity of the neural network involved in attention. Specifically, they found increased activity in the posterior cerebral lobule (lobule IV) and the superior parietal lobule following RehaCom in the treatment group only. A significant relationship was noted between behavioral gains post-treatment and increased activation in these brain regions, similar to others (50). Interestingly, lobule VI of the cerebellum is active in the articulatory control system; the authors thus concluded the increased activation noted in this region post-treatment to represent an increased effort to subvocally refresh subvocal stimuli in this system.

Sastre-Garriga et al. (83) found increased brain activity in the cerebellum following a treatment designed to target attention, speed of information processing, executive functions, memory, and higher level language processes. Participants who completed treatment demonstrated improvement in cognitive performance as well as increased brain activity in the anterior and posterior lobes of the right cerebellum. Although suffering from some methodological limitations, the authors were able to conclude that the positive impact of the cognitive rehabilitation on cognitive performance may, in fact, be mediated by increased activity within the cerebellum, a finding further supported by Cerasa et al. (53). Although the cerebellum is a largely understudied brain region as it relates to cognition and cognitive rehabilitation, it is important to note that two existing studies on cognitive rehabilitation in MS highlight the adaptive neuroplasticity of the cerebellum in response to a treatment for attention deficits. This is clearly a region ripe for future investigation.

Penner et al. (89) examined the effect of a 3- to 4-week computerized training program targeting selective attention in 11 patients with MS on patterns of cerebral activation on fMRI. Increased activation was seen post-treatment in MS patients with both mild and severe cognitive impairment in brain regions involved in attention, namely, the PCC, the precuneus, and the dorsal frontal cortex. Behavioral improvement correlated with the increased activation noted in these regions post-treatment. Although the lack of a control group in the study design was a limiting factor of this study, these data indicate that persons with MS can benefit from cognitive rehabilitation across the range of severity of cognitive impairment and neuroplasticity can be induced by cognitive rehabilitation procedures. Penner et al. (90) concluded that cognitive rehabilitation may enhance neuroplasticity in persons with MS and encourages the use of fMRI to enhance our understanding of the induced plasticity in persons with MS, as well as identify effective cognitive rehabilitation protocols.

**Figure 2:**
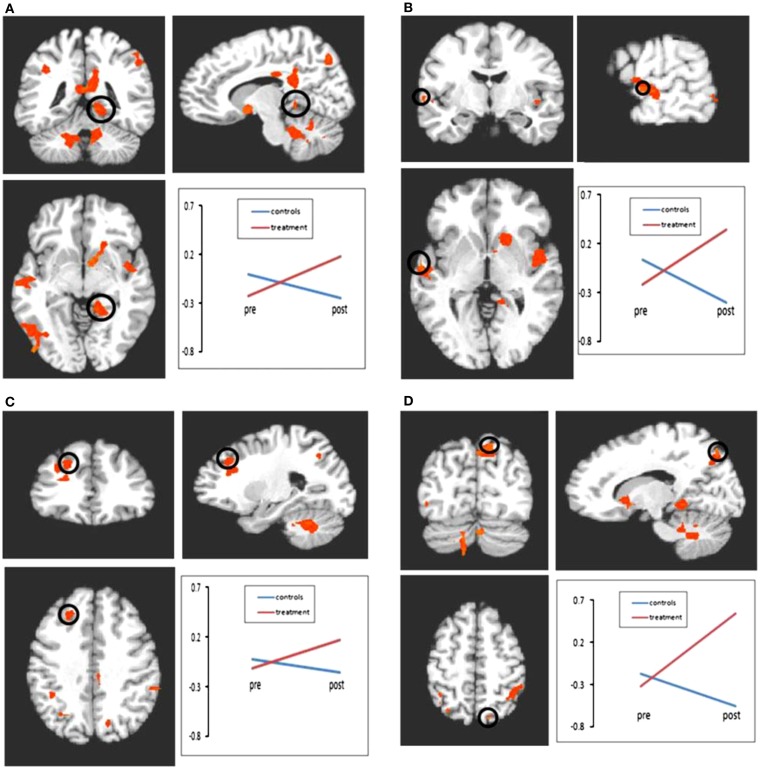
**Results of the 2 × 2 ANOVA with factors of time and group**. Following treatment, significant increases in activation were seen in the treatment group relative to the control group in regions including frontal lobe, parietal lobe, and cerebellum. All comparisons are significant at *p* < 0.01 (minimum cluster size = 10 voxels). **(A)** Bold activation change from pre- to post-treatment in parahippocampal gyrus. Control group represented by blue line; treatment group represented by red line. All interactions shown are significant at *p* < 0.01. **(B)** Bold activation change from pre- to post-treatment in superior temporal gyrus. **(C)** Bold activation change from pre- to post-treatment in middle frontal gyrus. **(D)** Bold activation change from pre- to post-treatment in precuneus. *Reprinted with permission from the Journal of Neurology.

**Figure 3:**
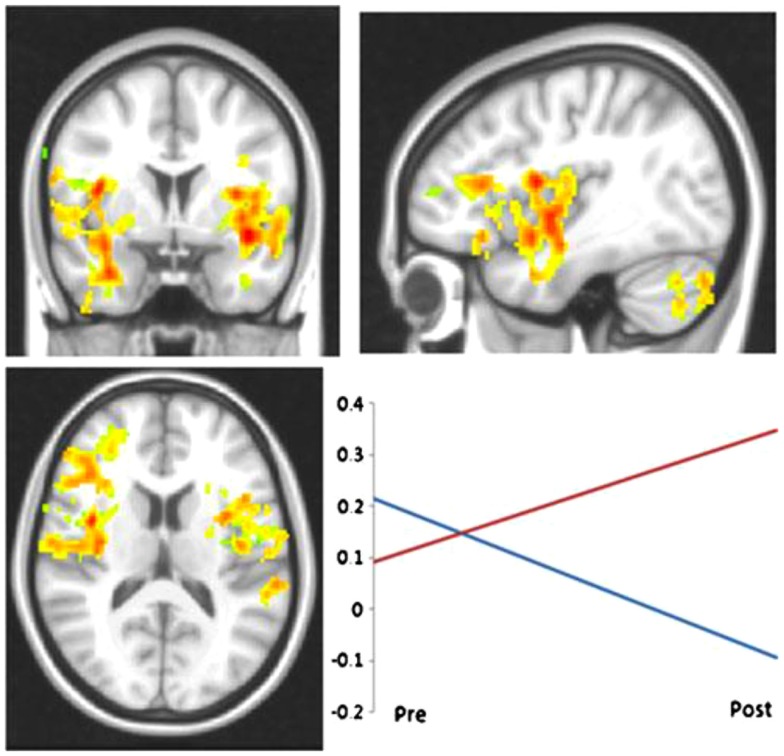
**LHIPP seed: increased connectivity between from LHIPP to left and right insula in the treatment group at post-treatment**. Interaction plot displays increased connectivity to left insula. *R*-values are plotted on the ordinate; time is plotted on the abscissa. Red line indicates treatment subjects, blue line indicates controls. *Reprinted with permission from Brain Imaging and Behavior.

Although limited in number, the two existing studies examining the cognitive rehabilitation of memory functioning in MS via neuroimaging techniques, also support the existence of induced neural plasticity in response to treatment. Chiaravalloti and colleagues utilized both fMRI (78) and FC (79) to evaluate a 10-session cognitive rehabilitation protocol specifically targeting new learning and memory abilities through a randomized clinical trial. After treatment, greater activation was evident only in the treatment group during performance of a memory task within a widespread cortical network involving frontal, parietal, precuneus, and parahippocampal regions (Figure 2). In a separate analysis by the same group (79), a significant increase in FC was noted in the treatment group post-treatment between the left hippocampus and cortical regions involved in memory functions, namely, the left insula, right parahippcampal gyrus, right insula, precentral gyrus, and post-central gyrus (Figure 3). These changes were not seen in the placebo-control group. These results demonstrate the neuroplasticity of the memory network in response to cognitive rehabilitation targeting learning and memory deficits in MS.

Ernst et al. (81) also examined the neuroplasticity associated with memory abilities, examining changes on fMRI following treatment focusing on autobiographical memories in an MS sample. The authors define autobiographical memory as the “capacity to relive detailed events, evoking spatiotempoal context, in which they were encountered as they are remembered.” The authors noted that following an intervention program for autobiographical memory, patients showed greater recruitment of the right cuneous, the left inferior and superior occipital gyri, the left precuneus and part of the lateral temporal cortex, largely on the left side, as compared with before treatment. These regions were consistent with regions known to be involved in the trained constructs. That is, changes were noted in posterior cerebral regions, known to be associated with the mental visual imagery trained in the training protocol applied. Significant improvement was noted in autobiographical memory, although the relationship between the behavioral and neuroimaging changes was not examined. Taken together, the two existing studies examining the efficacy of memory interventions in MS with neuroimaging both demonstrate increased activation in similar brain networks known to be integral to the trained function.

fMRI is thus a valuable tool to identify areas of dysfunction, and provides substantial evidence of both natural and induced neuroplasticity in persons with MS. Neuroplasticity in MS appears to largely be adaptive in nature when in response to rehabilitation, minimizing the clinical consequences of the neurological injury. It seems that a positive outcome of cognitive rehabilitation is likely the presence of post-treatment changes in fMRI, indicating the strengthening of existing regions and pathways associated with the treated domain. The application of neuroimaging measures to examine the functional and structural basis of changes in cognitive performance following cognitive rehabilitation will enhance our ability to identify the most effective treatments for persons with MS and modify such treatment to achieve maximal efficacy (91–93).

### Increased activation/connectivity: Adaptive or maladaptive?

Increases in cerebral activation, as well as increased functional connectivity can occur in persons with MS under varying conditions [see (94) for a complete discussion]. The first condition involves “local expansion.” The term “local expansion” refers to an increase in activation in the region immediately surrounding the lesioned area or area affected by the disease (95, 96). Specifically, persons in the early stages of MS have been shown to demonstrate increases in activation and connectivity, as compared with healthy controls, in the absence of cognitive impairment. Such changes in brain function are often associated with intact cognitive functioning and interpreted as adaptive neuroplasticity. For example, Forn et al. (70) demonstrated increased cortical recruitment during fMRI, reflecting the local expansion of activation, in cognitively preserved CIS patients (70) suggesting that early cortical changes may, in fact, limit the clinical expression of neuronal damage resulting from MS. Audoin et al. (69) similarly showed that CIS patients exhibited significantly greater activation in the regions *normally involved* in executive functioning: orbitofrontal regions, right cerebellum, and bilateral lateral prefrontal cortex (PFC) region during the PASAT, as compared with healthy controls, suggesting this activation to be adaptive. Amann et al. (97) examined the cerebral activation patterns associated with a working memory fMRI task in RRMS subjects with mild cognitive impairment and healthy controls and found that the overall pattern of brain activation was similar between the two groups. However, in the Anmann et al. study, persons with MS showed local expansion of cerebral activation during task performance within regions typically associated with working memory (i.e., anterior frontal and inferior parietal cortex). Similar findings were observed by Forn et al. (72) in an early RRMS sample. Taken together, these studies indicate that the maintenance of cognitive performance was due to the local cerebral expansion, interpreted as adaptive plasticity. Thus, in both CIS patients (69, 70) and patients with early RRMS (72, 97), there are indications of early plasticity of cognitive processes. While task performance is intact, the minimal additional recruitment typically seen in local expansion of activation appears to be an active and effective compensatory mechanism occurring early in the disease process.

A second condition is one in which we observe “homologous area adaptation.” Homologous area adaptation involves activation in areas in homologous regions of the contralateral hemisphere to the area impacted by disease (98, 99). In these instances, increases in cerebral activation/connectivity are correlated with *impaired* cognition. Chiaravalloti et al. (54) examined a modified PASAT administered via fMRI in three groups: MS with working memory impairment, MS without working memory impairment, and healthy controls. The healthy control group and MS group without working memory impairment showed a comparable activation pattern, i.e., primarily left hemisphere activation during working memory performance. However, in those MS individuals with working memory impairment, significantly more activation was noted bilaterally in the parietal and frontal regions in the MS group (indicative of both local expansion and homologous area adaptation). Further, the degree of extension of activation into the homologous right frontal region was correlated with *worse* cognitive performance, indicative of maladaptive neuroplasticity. This same pattern of results was also observed by Hillary et al. (55) on a different working memory task, with these authors interpreting their findings as indicative of neural inefficiency. Loitfelder et al. (100) compared HC with subjects with CIS, RRMS, and SPMS on a Go/No-Go task using fMRI and demonstrated that SPMS subjects showed activation in regions other than the task-related network observed in healthy controls (i.e., terms “extra-region” recruitment). The authors interpreted the observed extensive activation as neural inefficiency (100). Taken together, these several studies demonstrate cortical recruitment of “extra-regions” to support task completion, but this “extra-region” activation is associated with poorer cognitive functioning, and thus reflects maladaptive plasticity.

A final condition is one in which one observes increased activation in regions associated with the cognitive constructs being addressed within the treatment. This is precisely what is observed following cognitive rehabilitation. Multiple authors have shown increased activation of existing networks underlying trained functions in person with MS following treatment (78, 81). However, these areas are those known to underlie the performance of the skills taught during the active interventions. It thus appears that cognitive rehabilitation may not entail a traditional expansion of active brain regions into local or distal regions. In contrast, what appears to be occurring is increased activation of brain regions engaged by the techniques taught in treatment. This may be a strengthening of existing areas of activation or, in some cases, may involve newly activated regions. As an example, in Chiaravalloti et al. (78), increased activation was observed in the parietal regions during a verbal learning task. However, this activation is directly related to the techniques taught in treatment – visualization. This is thus activation supporting newly engaged cognitive processes that were shown to support the successful completion of the task.

In conclusion, increases in activation/connectivity are seen in persons with MS following cognitive rehabilitation and these increases are often associated with improvement in the targeted cognitive domain. While increased activation has been found with increased cognitive decline and disease progression in studies of the natural progression of MS, it is important to note that this increase in activation due to disease progression is distinct from the activation observed following cognitive rehabilitation. Thus, there are situations in which increased activation and/or connectivity is a negative consequence of the disease. In these situations, the activation we are observing might be best termed “maladaptive compensation.” That is, these extra areas of activation (or connectivity) are actually associated with worse performance and are therefore maladaptive (54, 55, 77). However, there are also situations in which such increases in activation and connectivity are positive, such as following effective cognitive rehabilitation. In these cases, the increased activation is associated with improvement in cognitive functioning and can thus be concluded to be adaptive. It is important to note that adaptive and maladaptive cerebral activation have been shown in the various disease stages (CIS, RR, SPMS). However, the documentation of cerebral reorganization via fMRI following cognitive rehabilitation has largely focused on RRMS patients to date. Thus, additional research is needed on cerebral reorganization after cognitive training, focusing on all MS phenotypes.

In reviewing the existing research, it is clear that there is significant neuroplasticity of cognitive functions in individuals with MS. Homologous region adaptation, local activation expansion, and extra-region recruitment all occur in an effort to maintain cognitive functioning. While much of this neuroplasticity is adaptive, it is important to note that in many situations, such neuroplasticity may be maladaptive, particularly in individuals that are demonstrating significant cognitive impairment and/or with disease progression. This maladaptive neuroplasticity (e.g., extra-region recruitment) may come at the cost of other cognitive functions for which the new areas now being utilized were crucial, such as processing speed. It is encouraging that such neuroplasticity can be induced through treatment such as cognitive rehabilitation, in an effort to “normalize” brain function and behavioral output. Moving forward, a focus on identifying adaptive versus maladaptive neuroplasticity associated with specific cognitive rehabilitation programs within all MS phenotypes would aide in the validation of the most effective cognitive rehabilitation interventions for persons with MS.

## Author Contributions

Dr. NC led the literature review, synthesis of the literature, and drafted the manuscript. Dr. HG assisted with synthesis of the literature and edited the manuscript several times. Dr. JD assisted the literature review, synthesis of the literature, and editing of the manuscript.

## Conflict of Interest Statement

Dr. Nancy D. Chiaravalloti reports no disclosures. Dr. Helen M. Genova reports no disclosures. Dr. John DeLuca has served as a consultant for Biogen IDEC and Novartis Pharmaceuticals. He has received grant funding from Biogen IDEC. He also is a journal club speaker for EMD Serono.
